# Fight against cholera outbreak, efforts and challenges in Malawi

**DOI:** 10.1002/hsr2.1594

**Published:** 2023-10-04

**Authors:** Mictum Miggo, Gracian Harawa, Allan Kangwerema, Simfukwe Knovicks, Chalo Mfune, Jackson Safari, John Thumbiko Kaunda, Joel Kalua, Glory Sefu, Elias Phiri, Parth Patel

**Affiliations:** ^1^ Clinical Department Queen Elizabeth Central Hospital Blantyre Malawi; ^2^ Clinical Department Mzuzu Central Hospital Mzuzu Malawi; ^3^ Department of Maternal Child Health Nkhatabay District Hospital Mzuzu Malawi; ^4^ Department of Veterinary Medicine University of Zambia Lusaka Zambia; ^5^ Department of Clinical Medicine Malawi College of Health Sciences Lilongwe Malawi; ^6^ Department of Monitoring and Evaluation Independent Monitoring and Evaluation Nairobi Kenya; ^7^ Department of Public Health, Graduate school of Health Sciences University of Ryukyus Nishihara Japan; ^8^ Department of Surgery, College of Surgeons of East Central and Southern Africa Arusha Tanzania; ^9^ The policy Unit Malawi Liverpool‐wellcome Trust Blantyre Malawi; ^10^ Public Health Department, School of Global and Public Health Kamuzu University of Health Sciences Blantyre Malawi; ^11^ Faculty of Public Health and Policy London School of Tropical Medicine London United Kingdom; ^12^ Clinical Department Atlas Medical Center Blantyre Malawi

**Keywords:** challenges, cholera, control, outbreak, prevention, response

## Abstract

Cholera is endemic in many African countries with recurrent seasonal outbreaks in parts of the region. Malawi has been experiencing seasonal outbreaks of Cholera since 1998, and it is one of the major public health problems. The current cholera outbreak is one of Malawi's worst cholera outbreaks in the past 10 years. Since the beginning of the outbreak about 56,090 cumulative cases of cholera have been reported with 1712 deaths representing a case fatality rate of 3.1%. This is happening when the country is recovering from the COVID‐19 epidemic, the devastating effects of tropical storms, and is also tackling the polio outbreak. Clearly, the Malawian health system is overstretched. Nevertheless, the country has taken a positive step in responding to the current cholera outbreak. Setting up treatment facilities, stepping up Water, Sanitation, and Hygiene (WASH) initiatives in impacted areas, and improving the surveillance system for early case detection and treatment are some of the actions taken. As the fight against cholera continues there is a need to significantly increase monitoring in all districts, particularly at the community level for early detection and control of the cholera. Considering there are some cross‐border cases from neighboring countries such as Mozambique, good collaboration between the two countries in strengthening surveillance and hygiene practices in the borders will help in controlling the spread of the disease. While it is commendable that dozens of oral cholera vaccines have been given, it should be noted that this provides short‐term prevention. In addressing the nation's ongoing and recurrent cholera outbreaks, we advise prioritizing WASH efforts in addition to oral cholera vaccine administration.

## 1 INTRODUCTION

Cholera remains a public health concern in Africa, especially in the sub‐Saharan region with many countries experiencing recurrent outbreaks.^1^ It is an acute diarrheal disease caused by the ingestion of water or food contaminated by the bacteria *Vibrio cholerae*. The disease manifests with acute watery diarrhoea which can be severe enough to cause severe dehydration and death.^2^, ^3^ Common drivers of cholera in Africa include insufficient water and sanitation facilities which sometimes are compounded by natural disasters.^4^ Recently the disease has been reported by several countries across Africa amidst the COVID‐19 pandemic posing a threat to already fragile health systems.^4^

Recently the disease has been reported by several countries across Africa amidst the COVID‐19 pandemic posing a threat to already fragile health systems.^4^ Between March 2022 and March 2023, 13 African countries experienced a cholera outbreak with a total of 145,121 reported cases with 3249 deaths; case fatality rate (CFR = 2.2%). Malawi has been reporting yearly seasonal outbreaks since 1998 with the southern parts mostly hit with recurrent outbreaks.^2^ At present, the country is struggling to stop the ongoing cholera outbreak which after months of devastating tropical storm Ana and cyclone Gombe displaced many people leaving them with poor access to safe water and sanitation facilities.^5^, ^6^ The current cholera outbreak is one of Malawi's worst cholera outbreaks in the decade.

## 2 CHOLERA BURDEN

The Malawi Ministry of Health (MOH) declared a cholera outbreak on March 3, 2022 following a case that was detected in the Machinga district which later saw the spread to other districts in the southern region.^5^ The outbreak has now spread to almost all parts of the country. MOH through the Public Health Institute of Malawi has been gathering surveillance data since the beginning of the current outbreak. The total cumulative cases are 56,090 since the outbreak started, according to a daily cholera bulletin released by the Ministry of Health on March 29, 2023. There have been roughly 1712 deaths reported across all affected districts in Malawi, representing a CFR of about 3.1%.^7^ This is a higher number than the reported cases in the neighboring countries. For example, Mozambique; 17,810 cases, 85 deaths (CFR = 0.5%), Zambia; 291 cases, 7 deaths (CFR = 2.4%), and Tanzania; 72 cases, 3 deaths (CFR = 4.2%).^8^

Among the most affected districts is Lilongwe district with the highest number of cases (12,226 and 548 deaths), Mangochi (8438 cases and 123 deaths), Blantyre (8167 cases and 211 deaths), Balaka (4278 cases and 102 deaths), Salima (3570 cases and 99 deaths), Machinga (2358 cases and 86 deaths), Dedza (2053 cases and 79 deaths), and Nkhatabay (1517 cases and 44 deaths) as of 29th March 2023.^9^ Most cholera cases are being diagnosed based on clinical symptoms and rapid antibody testing for *Vibrio cholerae*. There have been few cultures and genomic testing that have been conducted in a study setting. According to genomic evaluation, the majority of the cholera strain responsible for the outbreak in Malawi belonged to the ST69 seventh pandemic EL Tor (7PET) strain, with 80% expressing the 01 Ogawa serotype, which is a foreign strain not responsible for endemic cholera outbreaks in Malawi.^10^ The outbreak has been exacerbated by several risk factors, including disruptions to healthcare and Water, Sanitation, and Hygiene (WASH) infrastructure, limited access to WASH facilities in densely populated areas, and insufficient clinical care in peripheral facilities. Additionally, the displacement of communities due to cyclone storms further contributed to the lack of access to WASH facilities.^3^, ^5^, ^6^ These risk factors have also been observed in recent cholera outbreaks in Nigeria and the Democratic Republic of Congo, where inadequate WASH infrastructure and ongoing conflicts have also contributed to high cumulative cases of cholera.^8^

The current outbreak is happening at a time that Malawi's health system is overstretched as it is also fighting against rising polio cases. Therefore it is necessary to take organized measures to prevent the current cholera outbreak from spreading. The possibility of it reaching areas with limited access to clean water and sanitation facilities, especially in high‐risk districts, is a worrying issue.^9^

## 3 CURRENT EFFORTS

Following the devastating tropical storm which hit the southern region, Malawi moved quickly to map the high‐risk cholera areas to get ready for any cholera outbreak.^5^, ^6^ After the first case was confirmed and the outbreak declared, the Ministry of Health in coordination with World Health Organization (WHO), the United Nations International Children Education Fund (UNICEF), and other partners formulated a public health response plan to stop the disease.^5^, ^6^, ^7^, ^9^ This plan included several response activities such as awareness campaigns and strengthening water, sanitation, and hygiene in affected communities. Treatment units for cholera cases have been set in all affected areas and essential equipment and supplies, and diagnostic kits have been provided to ensure prompt case management and reduce mortality.

To mitigate the problem of water, and poor sanitation, MOH and UNICEF mobilized pot‐to‐pot chlorination of water and distributed the hygiene facilities such as buckets and soap in the affected communities.^5^, ^6^, ^9^ Treatment centers have also been equipped with prefabricated pit latrines and hand‐washing facilities. This is indeed a commendable effort in tackling the cholera outbreak. To enhance the surveillance system during the current outbreak, the MOH has mobilized Health Surveillance Assistants and community volunteers to support the surveillance, contact tracing, detection, and reporting of new cases.^5^, ^7^, ^9^ Community sensitization and awareness is being conducted in all parts of the country. This is being done with the help of community leaders who have been engaged to facilitate the construction of pit latrines and use by the members of the communities as well as formulating local by‐laws and action plans to stop the spread of cholera and encourage health‐seeking behaviors.^7^, ^9^

## 4 ORAL CHOLERA VACCINE ROLL OUT

Oral cholera vaccine has been shown to provide short‐term prevention against cholera and WHO advocates its use to complement the water and sanitation interventions.^5^, ^11^ In Malawi, the cholera vaccine was incorporated into the Malawi Cholera response plan in 2017.^12^ In response to the current outbreak, the country launched Oral cholera vaccination campaigns on May 23, 2022 to curb the further spread of cholera. This is being supported by Global Alliance for Vaccine and Immunization, and the global task force for cholera control. Global emergency stockpile has approved 3.9 million doses and has already provided Malawi with 1.9 million doses that target 8 priority districts that are deemed high risk for cholera.^5^, ^12^ It has been observed that cholera vaccine use in high‐risk areas has proven positive effect on cholera prevention and when integrated with water and sanitation interventions boosts the prevention and control of cholera.^6^, ^11^

## 5 CHALLENGES

Despite the rolling out of oral cholera vaccination campaigns, cholera cases continue to rise. One of the challenges and key drivers in the current outbreak is inadequate access to safe water, sanitation and hygiene facilities.^6^, ^7^ This has also been compounded by this year's tropical storm that damaged water supplies and left many people with limited access to safe drinking water. WHO advocates the WASH strategy with the provision of clean and safe drinking water, encouraging personal hygiene and also emphasizing household sanitation with a particular focus on human waste management.^2^ According to 2017 WASH statistics for Malawi, only 67% of the population had access to basic drinking water, 42% had access to basic sanitation and only 10% had basic hygiene services.^13^ This poses a threat to addressing cholera in the country. It is also further reported that of the people that use unimproved sources of water, 69% of that population does not treat their water before consumption and only 26% of that population uses an appropriate water treatment method.^13^ While there has been an increase in the percentage of Malawian households using water from an improved water source between 2004 and 2016, there is unequal distribution of such water sources with an estimated 30% of water points being non‐function at each point in the rural areas.^13^, ^14^ With these figures, it is apparent that Malawi still has a long way to go in increasing access to safe drinking water for all its population, a situation that is worrisome in the fight against cholera.

One important risk factor for frequent cholera outbreaks that the country must address is the issue of lack of proper human waste management. The 2015–16 Malawi Demographic Health Survey estimated an overall 17% of the population still practices open defecation and use of an unimproved shared toilet facility.^14^ The country's endeavor to curb frequent cholera outbreaks will be a success if human waste management becomes one of the key priorities in the cholera prevention strategy.^6^, ^14^ Another notable challenge that Malawi faces is its porous borders which allow easy cross‐migration as well as the importation of cholera cases. Cross‐border cholera outbreaks continue to be an important public health challenge in Sub‐Saharan African countries like Malawi.^13^ Malawi shares borders with three countries of Zambia, Mozambique, and Tanzania which poses a threat to the further spread of the outbreak. Previously the country has experienced cross‐border outbreaks along Mozambique and Tanzanian borders.^15^ The MOH reports that some of the cases of the ongoing cholera outbreak in Nsanje district were imported from Mozambique.^15^ Cross‐border movements of patients, insufficient risk assessment, and lack of information sharing and collaboration in implementing prevention activities between the two neighboring countries make the current cholera control difficult in the affected areas.^6^, ^15^

The surveillance system is one of the important cornerstones in any disease outbreak control. Early detection, reporting, and treatment of cases help to reduce the spread of the disease. In the present cholera outbreak, it has been noted that in some districts with high CFRs patients report late to the hospital owing to inadequate surveillance systems.^6^ This is worrisome as the epidemic continues to spread to more districts in the country. The current CFR of 3.1% is already higher than recommended CFR of 1% by WHO.

## 6 CONCLUSION AND RECOMMENDATIONS

Cholera is a major health concern in Malawi with one of the worst outbreaks in a decade. Measures like WASH interventions, case management, and vaccination campaigns are in place. However, poor hygiene, sanitation, cross‐border cases, and natural disasters pose challenges. To prevent future outbreaks, the government should invest in WASH interventions and promote behavior change for improved sanitation. Strengthening surveillance systems is also recommended.

## AUTHOR CONTRIBUTIONS

**Mictum Miggo**: conceptualization; project administration; supervision; writing—review & editing. **Gracian Harawa**: conceptualization; writing—review & editing. **Allan Kangwerema**: conceptualization; visualization; writing—original draft; writing—review & editing. **Simfukwe Knovicks**: resources; writing—original draft; writing—review & editing. **Chalo Mfune**: writing—original draft; writing—review & editing. **Jackson Safari**: resources; visualization; writing—review & editing. **John Thumbiko Kaunda**: writing—original draft; writing—review & editing. **Joel Kalua**: project administration; writing—review & editing. **Glory Sefu**: supervision; writing—review & editing. **Elias Phiri**: resources; supervision; writing—review & editing. **Parth Patel**: writing—original draft; writing—review & editing.

## CONFLICT OF INTEREST STATEMENT

The authors declare no conflicts of interest.

## TRANSPARENCY STATEMENT

The lead author Mictum Miggo affirms that this manuscript is an honest, accurate, and transparent account of the study being reported; that no important aspects of the study have been omitted; and that any discrepancies from the study as planned (and, if relevant, registered) have been explained.

## Data Availability

There is no data sharing applicable in this study as there are no datasets analyzed as it is a commentary study. No database or primary data was used in writing this paper.
